# Factors influencing the selection of an SGLT2i vs. a GLP-1RA as cardioprotective agent in patients with type 2 diabetes

**DOI:** 10.3389/fcvm.2025.1606198

**Published:** 2025-05-23

**Authors:** Shubham Agarwal, Mujeeb A. Basit, Michael E. Bowen, Daniel F. Heitjan, Christine Mai, Kelsea Marble, Jonathan Pak, Zichang Xiang, Ildiko Lingvay

**Affiliations:** ^1^ Division of Endocrinology, Department of Internal Medicine, The University of Texas Southwestern Medical Center, Dallas, TX, United States; ^2^ Division of Cardiology, Department of Internal Medicine and Clinical Informatics Center, The University of Texas Southwestern Medical Center, Dallas, TX, United States; ^3^ Division of General Internal Medicine, Department of Internal Medicine and Peter O’Donnell Jr. School of Public Health, The University of Texas Southwestern Medical Center, Dallas, TX, United States; ^4^ Peter O'Donnell Jr. School of Public Health, The University of Texas Southwestern Medical Center, Dallas, TX, United States; ^5^ Department of Statistics and Data Science, Southern Methodist University, Dallas, TX, United States; ^6^ The University of Texas Southwestern Medical Center, Dallas, TX, United States; ^7^ Boehringer Ingelheim Pharmaceuticals, Inc., Ridgefield, CT, United States; ^8^ Division of Endocrinology, Department of Internal Medicine and Peter O’Donnell Jr. School of Public Health, The University of Texas Southwestern Medical Center, Dallas, TX, United States

**Keywords:** SGLT2i, GLP-1RA, cardiovascular disease, type 2 diabetes, predictors

## Abstract

**Purpose:**

Sodium glucose co-transporter-2 inhibitors (SGLT2is) and glucagon-like peptide-1 receptor agonists (GLP-1RAs) have demonstrated cardioprotective effects in people with type 2 diabetes and atherosclerotic cardiovascular disease (ASCVD). In this patient group, there is treatment equipoise, from the standpoint of cardiovascular effect between these medication classes; however, factors associated with prescribing are poorly characterized.

**Methods:**

We performed a retrospective real-world analysis by creating an electronic health record registry of people with type 2 diabetes and ASCVD (without additional indications for a specific cardioprotective class) who received a prescription for either an SGLT2i or GLP-1RA. We analyzed patient-, provider-, and clinical encounter-related predictors of being prescribed an SGLT2i or GLP-1RA using univariable and multivariable logistic regression analysis.

**Results:**

A total of 573 eligible patients received either SGLT2i (*N* = 274) or GLP-1RA (*N* = 299) between January 2019 and October 2024. Care in cardiology (OR = 4.78; 95% CI, 2.53–9.04) strongly predicted SGLT2i prescription. Care in endocrinology (OR = 0.40; 95% CI, 0.23–0.68), higher BMI (OR = 0.92; 95% CI, 0.88–0.95, per BMI unit), and a higher recent estimated glomerular filtration (OR = 0.98; 95% CI, 0.96–0.99, per eGFR unit) predicted GLP-1RA prescription. The area under the receiver operating characteristic curve of the model was 0.78.

**Conclusion:**

Prescriber's specialty strongly determined the selection of cardioprotective agents. Treatment guidelines should provide more specific guidance regarding patient selection and consider the holistic benefits of each drug class beyond their cardiovascular protective effects.

## Introduction

Large cardiovascular outcome trials have demonstrated that sodium–glucose co-transporter-2 inhibitors (SGLT2is) and glucagon-like peptide-1 receptor agonists (GLP-1RAs) can reduce the risk of major adverse cardiovascular events (MACEs) among people with type 2 diabetes and pre-existing atherosclerotic cardiovascular disease (ASCVD) (1, 2).

Treatment guidelines for type 2 diabetes recommend the use of SGLT2i and GLP-1RA in this population to reduce the residual risk of MACE, irrespective of glycemic control or use of other glucose-lowering medications (3). Specifically, in people with ASCVD and type 2 diabetes without chronic kidney disease or heart failure, SGLT2is and GLP-1RAs have similar levels of evidence, and the guidelines do not provide a specific sequence of use or prioritization. Doing so disregards their differing mechanisms of action, additional effects of these drugs beyond MACE reduction, and consideration of patient factors. Furthermore, there are no randomized comparative effectiveness studies to inform the prescribing of SGLT2is or GLP-1RAs in this target population.

Because there is treatment equipoise between these two classes of cardioprotective agents among people with type 2 diabetes and ASCVD, we hypothesized that such selection might be driven by the presence of other comorbidities. We evaluated patient, provider, and health system characteristics associated with the selection of a cardioprotective agent.

## Methods

We performed a retrospective real-world data analysis using data extracted from the electronic health record (EHR) system (Epic Systems, Verona, WI, USA) of a tertiary-care, academic institution (University of Texas Southwestern Medical Center). The local Institutional Review Board approved the study.

Patients were eligible for the study if they had type 2 diabetes and ASCVD with at least one outpatient encounter in primary care, endocrinology, cardiology, or nephrology at which they were eligible for and prescribed either an SGLT2i or GLP-1RA. Encounters after 1 January 2019 (date of the first ADA treatment guidelines containing this recommendation) were included. Type 2 diabetes and ASCVD were defined using ICD-9/10 codes and SNOMED concepts from problem lists and encounter diagnoses. We excluded patients who received a prescription for both classes and patients with coexistent heart failure or chronic kidney disease stage 3 or higher as those conditions have indications for a specific cardioprotective class and are not in clinical equipoise.

Eligible patients were compiled into an EHR registry in October 2024 and grouped by the class of the first prescription, either an SGLT2i or GLP-1RA. Variables of interest at the prescription encounter included patient demographics (age, sex, ethnicity/race), medical information [BMI, systolic and diastolic blood pressure, family history of ASCVD, count of active medications, estimated glomerular filtration rate (eGFR), albumin-creatinine ratio, recent HbA1c, echocardiogram results, comorbidities (heart failure), recent diagnosis of ASCVD or type 2 diabetes, recent eligibility for either medication], encounter information [location (office or virtual), encounter duration, appointment type (new or established), initial encounter with specialty or provider, recent hospitalizations or emergency department visits], provider level-information [prescriber specialty, type of provider (attending physician, mid-level provider, trainee physician), provider years of experience, previous providers] and health system-related variables (health insurance: commercial, Medicare, other).

We used logistic regression to create a statistical model predicting the prescription outcome from baseline variables. We conducted univariate and multivariate analyses. For the univariate analysis, we included each variable separately as the sole predictor in a logistic model. In the multivariate analysis, we used stepwise regression to identify the best predictive model. We forced three variables—age at prescription, ethnicity/race, and sex—into the final model regardless of their statistical significance, as we wished to evaluate the influence of these variables. For both univariate and multivariate analyses, we report the odds ratio, 95% confidence interval for the odds ratio, and *p*-value for each predictor. We present a receiver operating characteristic (ROC) curve and report the area under the ROC curve (AUC) for the final model. We assessed the model's goodness of fit by the Hosmer–Lemeshow test (4). We evaluated collinearity of predictors by computing the variance inflation factor, which is the ratio of the variance of a predictor in the multivariate model to its variance in a univariate model. We conducted all statistical analyses in SAS version 9.4 (Cary, NC, USA; SAS Institute, Inc.).

## Results

We identified 17,740 patients with type 2 diabetes and cardiovascular and/or kidney disease in whom guideline-directed medical therapy included either an SGLT2i or GLP-1RA. Of the 3,319 patients with type 2 diabetes and ASCVD without chronic kidney disease stage 3 or higher or heart failure, 573 (17%) patients received a guideline-directed prescription for either an SGLT2i (*N* = 274) or a GLP-1RA (*N* = 299). The proportion of prescriptions for SGLT2is vs. GLP-1RAs varied over the study period, with GLP-1RAs being predominant in the last 2 study years (Supplementary Figure S1). The characteristics of the two groups are presented in Table 1. Supplementary Table S1 shows the median time from the time of prescription to when the most recent prior laboratory tests, vital signs, and BMI were obtained. Those prescribed a GLP-1RA were predominantly female, on average younger, and had a higher BMI than those who received an SGLT2i.

**Table 1:** Baseline characteristics of the included people with type 2 diabetes and ASCVD who were prescribed a GLP-1RA or SGLT2i displayed by medication class prescribed.

Characteristic	Prescribed GLP-1RA (*N* = 299)	Prescribed SGLT2i (*N* = 274)	*p*-value
Age, years	65 ± 10.1	68.8 ± 9.4	<0.01
Gender, female	123 (41.1)	82 (29.9)	<0.01
Race/ethnicity			0.28
Hispanic/Latino	53 (17.7)	45 (16.4)	
Non-Hispanic Asian	17 (5.7)	29 (10.6)	
Non-Hispanic White	141 (47.2)	126 (46.0)	
Non-Hispanic Black	69 (23.1)	54 (19.7)	
Non-Hispanic Other	21 (7.0)	20 (7.3)	
BMI, kg/m^2^	33.7 ± 6.7	30 ± 5.8	<0.01
SBP, mmHg	132.7 ± 17.8	134.5 ± 19.7	0.24
DBP, mmHg	77.4 ± 8.7	76 ± 9.1	0.06
HbA1c, %	7.8 ± 1.9	7.5 ± 1.5	–
eGFR, ml/min/1.73 m^2^	72.1 ± 15.2	68.6 ± 13	<0.01
Height, inches	67.3 ± 4.5	67.4 ± 4.1	0.87
Weight	3,479.6 ± 789.1	3,106.9 ± 691.1	<0.01
ACR >30	55 (18.4)	50 (18.2)	0.96
Family history of CAD	84 (28.1)	92 (33.6)	0.13
Number of eligible encounters in the prior year	3.5 ± 2.6	3.3 ± 2.5	0.49
Encounter type			0.40
Office visit	256 (85.6)	244 (89.1)	
Video visit	44 (14.7)	30 (10.9)	
House visit	1 (0.3)	0 (0)	
New diagnosis of ASCVD	2 (0.7)	3 (1.1)	0.59
Hospital admission in the prior year	37 (12.4)	46 (16.8)	0.14
ED visit in the prior year	26 (8.7)	10 (3.6)	0.02
New HbA1c result	29 (9.7)	14 (5.1)	0.04
New LVEF result	0 (0)	0 (0)	1.00
New eGFR or ACR result	33 (11.0)	22 (8.0)	0.23
New to clinic	66 (22.1)	53 (19.3)	0.54
New presentation to provider	115 (38.5)	81 (29.6)	0.03
New to specialty	53 (17.7)	42 (15.3)	0.51
New eligibility	77 (25.8)	78 (28.5)	0.41
First eligible visit to specialty	108 (36.1)	94 (34.3)	0.78
Encounter specialty			<0.01
Cardiology	21 (7.0)	101 (36.9)	
Endocrinology	155 (51.8)	66 (24.1)	
Primary care	125 (41.8)	104 (38.0)	
Encounter provider			0.03
Trainee physician	19 (6.4)	13 (4.7)	
Advanced practice provider	73 (24.4)	41 (15.0)	
Independent physician	201 (67.2)	218 (79.6)	
Established to clinic	235 (78.6)	221 (80.7)	–
Primary insurance type			0.17
Commercial	108 (36.1)	78 (28.5)	
Medicare	188 (62.9)	193 (70.4)	
Exchange	1 (0.3)	0 (0)	
Appointment duration			<0.01
20 min	95 (31.8)	130 (47.4)	
30 min	89 (29.8)	64 (23.4)	
40 min	87 (29.1)	63 (23.0)	
60 min	17 (5.7)	16 (5.8)	
>60 min	11 (3.7)	11 (4.0)	

*p*-values are from univariate logistic regression analyses. Continuous variables are presented as mean with standard deviation. Categorical variables are presented as count and percentage. ASCVD, atherosclerotic cardiovascular disease; SGLT2i, sodium glucose co-transporter-2 inhibitors; GLP-1RA, glucagon-like peptide-1 receptor agonists; BMI, body mass index; SBP, systolic blood pressure; DBP, diastolic blood pressure; HbA1c, hemoglobin A1c; eGFR, estimated glomerular filtration rate; ACR, albumin-creatinine ratio; CAD, coronary artery disease; ED, emergency department; LVEF, left ventricular ejection fraction.

In the final model, age, sex, and ethnicity/race were not statistically significant; the only significant predictors retained were prescriber's specialty, BMI, most recent eGFR, and appointment duration (Table 2). Receiving care at the cardiology clinic (OR 4.78, 95% CI 2.53–9.04, *p* < 0.001) in comparison with primary care or nephrology was a strong predictor of being prescribed an SGLT2i. Factors that predicted greater likelihood of a GLP-1RA prescription included receiving care at the endocrinology clinic (OR = 0.40, 95% CI 0.23–0.68, *p* < 0.001) in comparison with primary care or nephrology clinic, higher BMI (OR = 0.92, 95% CI 0.88–0.95, *p* < 0.001), and a higher most recent eGFR (OR = 0.98, 95% CI 0.96–0.99, *p* = 0.002). Shorter appointment times (20, 30, or 40 min vs. 60 min reference) predicted prescription of an SGLT2i (*p* = 0.033); although individual contrasts compared with the reference of 60 min were not significant, the contrast comparing averages in the two shorter categories to the two longer categories gave an OR of 2.03 (95% CI 1.17–3.50, *p* = 0.012).

**Table 2:** Multivariable analysis conducted using logistic regression with age, ethnicity/race, and sex forced into the final model regardless of their statistical significance.

Variables	Odds ratio	95% CI	*p*-value
Age, per year	1.022	(0.999, 1.046)	0.063
Ethnicity/race (non-hispanic White reference)			0.734
Hispanic or Latino	0.940	(0.525, 1.681)	
Non-Hispanic Asian	1.310	(0.591, 2.905)	
Non-Hispanic Black	0.972	(0.564, 1.672)	
Non-Hispanic Other	1.641	(0.713, 3.776)	
Sex, male reference	0.763	(0.493, 1.180)	0.223
BMI (per BMI unit)	0.915	(0.881, 0.951)	<0.001
Most recent eGFR (per eGFR unit)	0.977	(0.962, 0.991)	0.002
Specialty (reference primary care)			<0.001
Cardiology	4.784	(2.532, 9.039)	
Endocrinology	0.396	(0.232, 0.677)	
Appointment duration (reference 60 min)			0.033
20 min	1.961	(0.759, 5.068)	
30 min	2.128	(0.818, 5.541)	
40 min	1.017	(0.376, 2.748)	

Data are presented as odds ratio, 95% confidence intervals for the odds ratio, and *p*-value for each predictor. BMI, body mass index; eGFR, estimated glomerular filtration rate.

The multivariable model was judged to fit well (*p* = 0.45 by the Hosmer–Lemeshow test). Multicollinearity was modest, with variance inflation factors ranging from 1.04 to 1.35.

**Figure 1:**
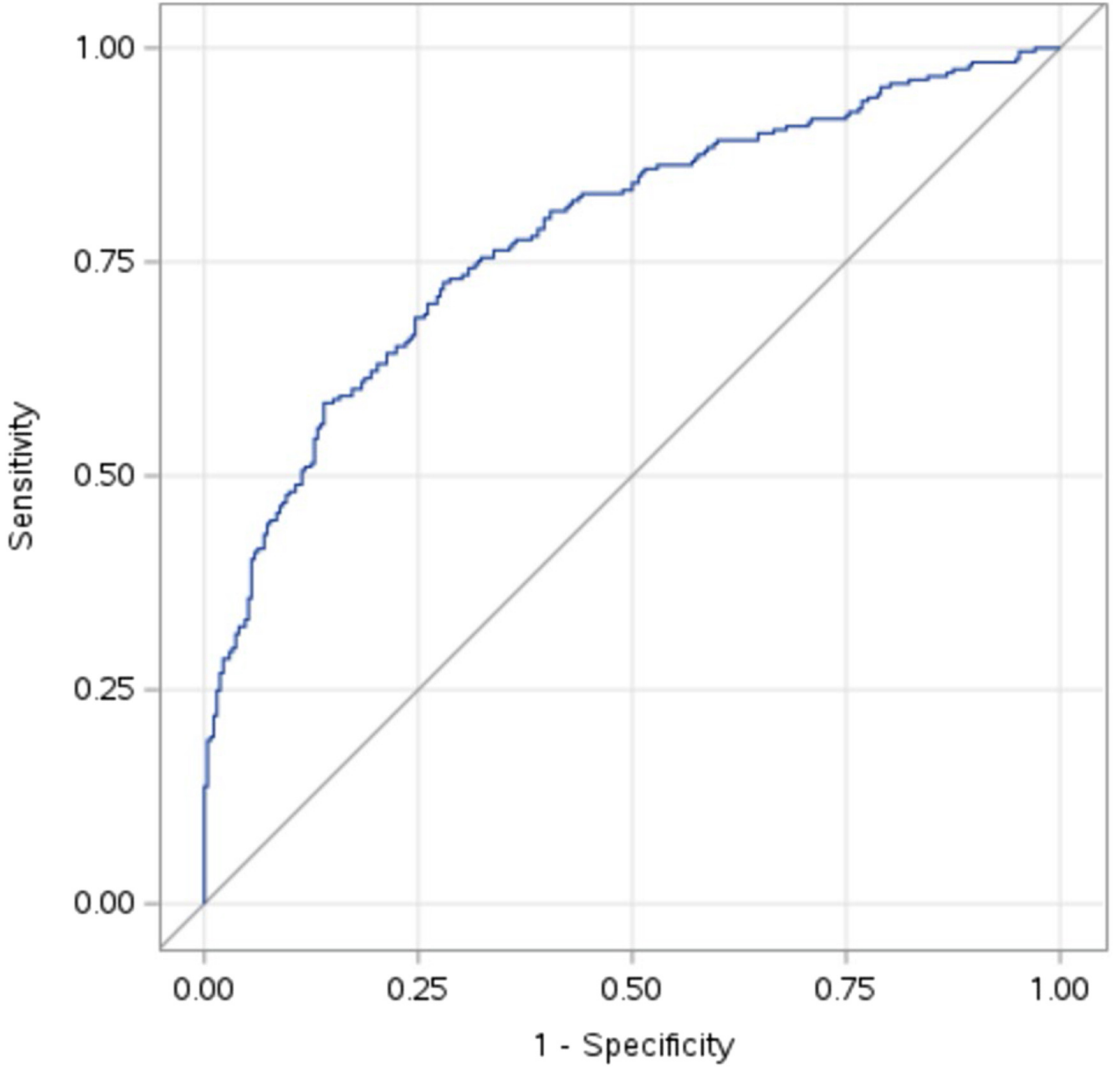
Receiver operating curve for the logistic regression model with an area under the curve of 0.78.

The model predicted the outcome well, with an AUC of 0.78 (Figure 1).

## Discussion

We found that under clinical equipoise, the prescription of SGLT2i vs. GLP-1RA among people with type 2 diabetes and ASCVD was significantly influenced by the specialty of the prescribing provider, and to a lesser degree by BMI, most recent eGFR level, and appointment duration. Cardiologists were much more likely to prescribe SGLT2i, while endocrinologists were much more likely to prescribe GLP-1RA. Higher BMI and higher most recent eGFR had a significant but weaker association with prescription of GLP-1RA, and shorter appointment duration was weakly associated with prescription of SGLT2i.

The strong specialty preference for a specific class is notable. Possible reasons for the strong preference for SGLT2i among cardiologists include its ease of use, absence of titration requirement, oral medication formulation, and their familiarity with SGLT2i, considering its recommended use in heart failure (5). In contrast, the recognition of therapeutic weight loss as a pillar of cardiometabolic disease treatment and familiarity with the use of GLP-1RA since its initial approval (as a glucose-lowering agent) might influence endocrinologists to preferentially prescribe GLP-1RA (6). Other studies have shown a larger prescription gap of cardioprotective agents among cardiologists than endocrinologists (7, 8), although these did not specifically look at the population with type 2 diabetes and ASCVD, where there is clinical equipoise with respect to cardioprotective medication. Data from these studies, including ours, can help tailor specialty-directed education programs to enhance guideline-based prescribing while addressing practitioner biases.

GLP-1RAs are known to facilitate weight loss, and the likelihood of people with higher BMI receiving a GLP-1RA is expected (9). Preferential use of SGLT2i in people with lower eGFR could be driven by its recommended use of in people with CKD or the anecdotal concerns of increased gastrointestinal adverse events with GLP-1RA in people with low eGFR. However, none of the included patients in the study had CKD stage 3 or above at eligibility. The association of shorter appointment duration with SGLT2i prescription may be the result of greater complexity and, therefore, provider time required to initiate a GLP-1RA, which requires patient education regarding prevention and management of gastrointestinal side effects, injection technique, and dose titration. While some assessed variables (i.e., age and sex) were significant in the univariable models, the final model (which had a robust ROC) contained only a small number of predictors, likely due to the overwhelming effect of the prescribers’ specialty.

Our study evaluated a unique group of people in treatment equipoise regarding the choice of cardioprotective agents. Previous studies assessing predictors of the selection between the two classes evaluated broader population groups who were not at treatment equipoise. A retrospective analysis of a random sample of prescriptions from hospitals in China in the general population showed that older adults, of male gender, without health insurance, and those seen in cardiology clinics were more likely to be prescribed an SGLT2i than a GLP-1RA (10). Similarly, a study analyzed the prescription of cardioprotective agents vs. no prescription in a cohort of underrepresented ethnicities in the US with type 2 diabetes (11). People with higher BMI, of female sex, younger age, with higher income, having health insurance coverage, of Hispanic ethnicity, and non-Hispanic black race were more likely to receive a GLP-1RA (vs. not receiving GLP-1RA). People with higher BMI, of male sex, of Hispanic ethnicity, non-Hispanic black race, and Asian race were more likely to receive an SGLT2i (vs. not receiving SGLT2i). In contrast, our study shows the likelihood of being prescribed one class over the other when equally eligible for either class according to the current guideline indications for the population evaluated.

Our study findings indicate that, under clinical equipoise for a cardiovascular indication, the selection of these medication classes is largely influenced by specialty preferences, with lesser consideration for patient-specific factors or the distinct mechanisms of action between the two drug classes. This approach does not optimize all aspects of patient care. Treatment guidelines should provide objective criteria for selecting a drug class based on the holistic effects of these drugs beyond just their effect on MACE. Additionally, our results could help inform quality improvement efforts to increase specialty-driven guideline-indicated prescribing patterns and create a framework for future studies aimed at investigating how prescriber specialty influences patient outcomes. However, it does not address patient-related barriers to filling such prescriptions or long-term adherence and persistence on therapy. We were also unable to account for drug intolerance, allergies, and other contraindications (e.g., out-of-pocket cost) that might discourage healthcare providers from prescribing cardioprotective medications; however, these would not be expected to differ considerably between specialties. The study was conducted at a single tertiary-care hospital which may not represent the broader population. At the same time, our study included a large dataset, evaluated a large number of potential clinical predictors, and is unique in examining predictors at the encounter level. Furthermore, our dataset is the most contemporaneous (data up to October 2024), reflecting current practice patterns.

## Conclusions

In people with type 2 diabetes and ASCVD who have an equal indication for either SGLT2i or GLP-1RA for cardioprotection, selection between these classes was strongly determined by the prescriber's specialty and, to a lesser degree, by the person's BMI, most recent eGFR, and appointment duration time. These findings indicate high specialty-specific biases when prescribing cardioprotective medications.

## Data Availability

The original contributions presented in the study are included in the article/Supplementary Material, further inquiries can be directed to the corresponding author.
