# Supplementary material for: Factors influencing the selection of an SGLT2i vs. a GLP-1RA as cardioprotective agent in patients with type 2 diabetes
Source: Front Cardiovasc Med. 2025 May 23;12:1606198. doi: 10.3389/fcvm.2025.1606198 (PMC12141205; doi:10.3389/fcvm.2025.1606198)
Supplement: Supplementary file 1 [file Datasheet1.pdf]

Manuscript title:

Factors influencing the selection of an SGLT2i vs a GLP-1RA as cardioprotective agent in patients with type 2 diabetes

Supplementary File

Tables

Table S1: Median time from the encounter in which the medication was prescribed to the most recent prior patient variable of interest extracted from the electronic health record.

Patient variables	Median	IQR
Time since most recent HbA1C (days)	94.1	157.3
Time since most recent Height (days)	0.0	0.0
Time since most recent Weight (days)	0.0	0.0
Time since most recent BMI (days)	0.0	0.0
Time since most recent Diastolic Blood Pressure (days)	0.0	0.0
Time since most recent Systolic Blood Pressure (days)	0.0	0.0
Time since most recent eGFR (days)	49.0	133.8

Abbreviations used: IQR = interquartile range, HbA1c = Hemoglobin A1c, BMI = Body-mass index, eGFR = estimated glomerular filtration rate

Figures

Figure S1: Temporal trends of the proportion of SGLT2i vs GLP-1RA prescriptions over the duration of the study

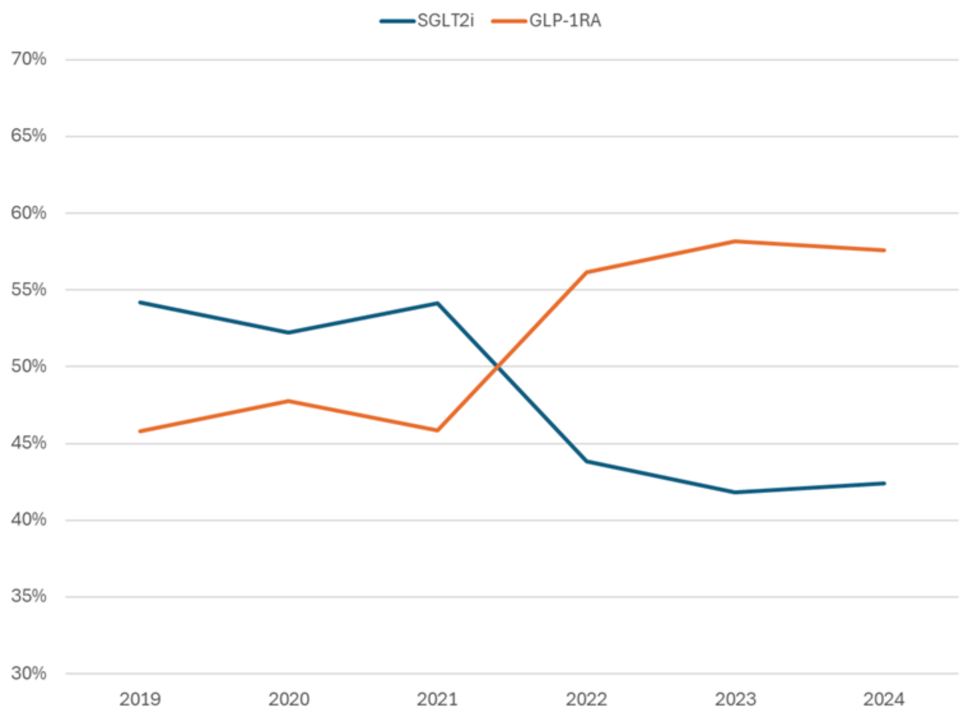

Appointment Year	2019	2020	2021	2022	2023	2024 (through Oct)
SGLT2i	45	35	59	50	46	39
GLP-1RA	38	32	50	64	64	53
